# Towards a Quantitative Single Particle Characterization by Super Resolution Microscopy: From Virus Structures to Antivirals Design

**DOI:** 10.3389/fbioe.2021.647874

**Published:** 2021-03-26

**Authors:** Maria Arista-Romero, Silvia Pujals, Lorenzo Albertazzi

**Affiliations:** ^1^Nanoscopy for Nanomedicine Group, Institute for Bioengineering of Catalonia (IBEC), The Barcelona Institute of Science and Technology, Barcelona, Spain; ^2^Department of Electronics and Biomedical Engineering, Faculty of Physics, Universitat de Barcelona, Barcelona, Spain; ^3^Department of Biomedical Engineering, Institute for Complex Molecular Systems (ICMS), Eindhoven University of Technology, Eindhoven, Netherlands

**Keywords:** super-resolution, virus, characterization, antivirals, imaging

## Abstract

In the last year the COVID19 pandemic clearly illustrated the potential threat that viruses pose to our society. The characterization of viral structures and the identification of key proteins involved in each step of the cycle of infection are crucial to develop treatments. However, the small size of viruses, invisible under conventional fluorescence microscopy, make it difficult to study the organization of protein clusters within the viral particle. The applications of super-resolution microscopy have skyrocketed in the last years, converting this group into one of the leading techniques to characterize viruses and study the viral infection in cells, breaking the diffraction limit by achieving resolutions up to 10 nm using conventional probes such as fluorescent dyes and proteins. There are several super-resolution methods available and the selection of the right one it is crucial to study in detail all the steps involved in the viral infection, quantifying and creating models of infection for relevant viruses such as HIV-1, Influenza, herpesvirus or SARS-CoV-1. Here we review the use of super-resolution microscopy (SRM) to study all steps involved in the viral infection and antiviral design. In light of the threat of new viruses, these studies could inspire future assays to unveil the viral mechanism of emerging viruses and further develop successful antivirals against them.

## Introduction

From the discovery of viruses by Dmitri Ivanowsky and Martinus Beijerinck in 1892 and 1898, respectively (Artenstein, 2012), viral characterization has become a key element to understand and fight viral infections and pandemics such as the Spanish Flu in 1918, the HIV-1 infection in the 80’s and the COVID-19 in 2020. Viruses have been intensely studied with biochemical techniques such as ELISA, Western blots, PCRs and hemagglutination assays to understand the protein composition and the affinity toward antivirals and target cells (Vajda et al., 2016; Ustinov et al., 2017; Rumlová and Ruml, 2018). Nevertheless, these techniques measure viral particles in bulk at the same time, leading to a lack of understanding of protein structures and the spatial distribution of the viral proteins within the particle (Payne, 2017; Guliy et al., 2019). Microscopy contributes to fill this gap by studying structure and function of viruses in space and time. Fluorescence microscopy in particular allows visualization of multiple viral proteins thanks to its multicolor ability, and is in fact one of the main tools of modern virology (Nathan and Daniel, 2019; Carravilla et al., 2020). However, due to the small size of viruses, usually smaller than 200 nm, conventional fluorescence techniques are lacking sufficient resolution power to resolve individual viral structures. To overcome this knowledge gap, single-particle studies have been suggested to study viral features individually (Kiss et al., 2020).

Single-particle measurements allow the individual characterization of viral particles, analyzing surface composition, interactions and forces of individual viral structures. The conventional single-particle techniques up to date are optical tweezers, atomic force microscopy (AFM) and electron microscopy (EM), each of them with their advantages and disadvantages. Optical tweezers is a single-molecule technique that allows the measurement of attracting and repulsing forces between two samples or between the molecule and the environment. In virology, it has been used to study the packaging of the DNA of bacteriophages (Smith D.E. et al., 2001; Liu et al., 2014), to characterize the viral assembly of Simian virus 40 (SV40) (van Rosmalen et al., 2020), to identify the integrity and interaction of an anti-HIV-1 enzyme (APOBEC3G) (Morse et al., 2019) and to study the stability of the hairpin of the RNA of HIV-1 virus while is interacting with Gag protein (McCauley et al., 2020). Despite their potential, optical tweezers are very restricted in the type of sample that they can hold; trapping nanostructures such as nanoparticles is still difficult and imprecise (Dhakal and Lakshminarayanan, 2018), thus most viral studies have focused their attention to DNA, proteins and certain isolated structures.

Atomic force microscopy scans the surface of the viral particle and can identify surface viral structures, conducting forces and force measurements between the tip and the surface. This technique has been reported in detail in the characterization of viral systems (Baclayon et al., 2010; Kuznetsov and McPherson, 2011; Marchetti et al., 2016; de Pablo and Schaap, 2019) with positive results, such as the characterization of the forces of interaction between influenza viruses and their host cells (Sieben et al., 2012), the surface ultrastructure characterization of the SARS-CoV 1 and herpesvirus (Lin et al., 2005; Dou et al., 2020) or monitoring of retroviral budding process (Gladnikoff and Rousso, 2008); but AFM possesses several limitations such as limited scan size (several microns square), a delicate tip that can be damaged easily, a slow scanning time and the analysis which due to a lack of labeling, is only limited to surfaces, hence specific structures cannot be identified (Sinha Ray, 2013).

Lastly, from the first image of a virus obtained in 1938 and 1939 (von Borries et al., 1938; Ruska et al., 1939), EM, transmission electron microscopy (TEM), scanning electron microscopy (SEM) and cryo-electron microscopy (cryo-EM) have been the main microscopy techniques used to study and characterize viruses (Leser and Lamb, 2017), virus-like particles (McCraw et al., 2018), viral glycoproteins (Wu and Wilson, 2020) and viral inhibitors (Liu et al., 2020; Zhou et al., 2020). However, traditional morphological studies require the negative staining of the sample under extreme conditions. Due to the exposure of the sample to heavy metals, non-physiological pH, dehydration and sectioning, the shape and morphology of the virus particle can suffer modifications and artifacts, leading to irregular virus shapes and broken structures (Mollenhauer, 1993; Ayache et al., 2010; Gulati et al., 2019). These drawbacks were overcome by cryo-EM, a technique that fixes the sample by freezing. However, this is a quite delicate process that also could generate artifacts (Passmore and Russo, 2016).

Despite the individual disadvantages, in general the main drawback of these single-particle techniques is the lack of multicolor characterization, 3D imaging and live-cell tracking information that can be obtained with fluorescence probes. Related studies have been done using confocal microscopy focusing mainly on how infected cells respond to infections and antivirals (Howell and Miller, 1999; Lorin et al., 2020), but since viruses are smaller than 200 nm, it is impossible to study the virus architecture using conventional fluorescence microscopes with immunostaining; this is why SRM techniques are needed to understand and quantify viral protein distribution and cell-virus interaction.

Super-resolution microscopy is a family of different microscopic techniques that embrace the same goal: to break the diffraction limit of the light, described by Abbe in 1873 (Abbe, 1873), to achieve resolutions between 1 and 100 nm (Gwosch et al., 2020). The whole family of SRM methods are based on different principles and instruments (Schermelleh et al., 2010). With these specific characteristics, three main types of SRM can be found: stimulated emission depletion (STED) (Hell and Wichmann, 1994), structured illumination microscopy (SIM) (Gustafsson, 2000) and single molecule localization microscopy (SMLM) (Betzig et al., 2006; Hess et al., 2006; Rust et al., 2006; Sharonov and Hochstrasser, 2006; Sahl et al., 2017; Pujals et al., 2019).

• Stimulated emission depletion microscopy achieves resolutions of 20–80 nm reducing the size of the point-spread function (PSF) by illuminating the sample with two beams aligned: a conventional excitation beam (to excite the fluorophore) and a depletive doughnut-shape laser (to deplete the excitation on the doughnut area). In this way, only the fluorophores located in the center of the doughnut will emit fluorescence, reducing the PSF to a narrower area and thus improving the final resolution (Klar and Hell, 1999; Göttfert et al., 2013; Vicidomini et al., 2018).
• Structured illumination microscopy allows super-resolution by using structured patterns of illumination of light across the sample. When the sample contains features smaller than the diffraction limit, the patterned illumination applied creates an interference pattern (Moiré fringes). By changing the position and orientation of the structured shape, several Moiré fringes are created that can be mathematically deconvolved, producing a high-resolution image with a resolution of 100 nm (Wu and Shroff, 2018).
• Single molecule localization microscopy includes three super-resolution techniques: Stochastic Optical Reconstruction Microscopy (STORM), Photoactivated Localization Microscopy (PALM) and Points Accumulation for Imaging in Nanoscale Topography (PAINT). In general, SMLM consists in the localization of the center of emission of the probe by stochastic cycles of transient emission of the fluorophores of the sample. A “blinking” is generated when the probes switch from an “emission” state to a “dark” state individually, as a result, single fluorophores can be detected by fitting a Gaussian profile, which identifies the spatial positioning of all the emitting molecules of the sample, reconstructing the super-resolution image (Sauer and Heilemann, 2017). The differences between STORM, PALM, and PAINT rely on the nature of the blinking. While STORM and PALM are based on photo-switchable probes and photo-activable proteins respectively, PAINT employs labeled probes with a transient binding to targeted molecules (Sharonov and Hochstrasser, 2006; Jungmann et al., 2010, 2014; Lew et al., 2011; Schnitzbauer et al., 2017; Oi et al., 2020).

The most outstanding differences between the SRM techniques described are summarized in Table 1. We want to highlight the final resolutions achieved in SRM, oscillating between 10 and 50 nm in most cases. Contrarily, the most noticeable disparity is the acquisition times, which could fluctuate to few seconds using SIM and to several minutes with PAINT. Finally, the distinctive technical differences between the SRM techniques are explained in detail in several reviews previously published, which describe intensively the strength and weakness of each microscopy technique (Thorley et al., 2014; Sahl et al., 2017; Huszka and Gijs, 2019).

**TABLE 1:** Resume of the main super-resolution microscopy techniques. STORM, PAINT, PALM, and their derivate can be classified as single-molecule localization microscopy (SMLM).

Name	Resolution	Probes	Illumination method	Acquisition time	Main advantage	References
SIM	∼100 nm	Conventional fluorescence probes	Wide-field TIRF (optional)	Short (seconds)	Fast, live-cell imaging and 3D	Gustafsson, 2000; Young et al., 2016
STED	20–80 nm	Special photostable dyes –Emission spectra overlaps depletion wavelength	Laser scanning confocal	Short (seconds)	Fast and good resolution without post-processing	Hell and Wichmann, 1994
**SMLM**						
STORM/dSTORM	10–55 nm	Photo-switchable dyes	Wide-field TIRF	Long (minutes)	Good axial and lateral resolution with normal immunostaining	Rust et al., 2006
PALM/FPALM	10–55 nm	Photo-activable fluorescence proteins	Wide-field TIRF	Long (minutes)	Good axial and lateral resolution with fluorescence proteins from the sample	Betzig et al., 2006; Hess et al., 2006
iPALM	∼20 nm	Photo-activable fluorescence proteins	Wide-field TIRF	Long (minutes)	Allows performing 3D images with high accuracy	Shtengel et al., 2009
PAINT/DNA-PAINT	5–25 nm	Binding probes with specificity attachment	Wide-field TIRF	Long (minutes)	Multicolor-imaging (up to 124) Quantification	Sharonov and Hochstrasser, 2006; Jungmann et al., 2010; Schnitzbauer et al., 2017

There are several advantages of using SRM and one of them is the ability to perform multicolor imaging, something impossible to achieve with the single-particle techniques previously explained. By multicolor acquisition it is possible to characterize the spatial distribution of 2 or more proteins of the virus along the viral particle (Lehmann et al., 2011, 2016). Moreover, the main leading feature of SMLM is the possibility to quantify the amount of labeled proteins that are in the analyzed area, performing multicolor-quantifications at single particle level. This opens the possibility to measure the amount of proteins clustered in certain areas of the viral particle and infected cell membrane and to analyze how it changes with time, like the study performed by Gunzenhäuser et al. (2012) on HIV-1.

Next, live-cell imaging is still a challenge for some SRM methods but in many cases it could be done with the correct settings of the microscope, adjusting the acquisition times and light power to avoid cell damage and monitor the movement of viral-particle trafficking (Wäldchen et al., 2015). In this sense STED and FPALM have been improved to allow the live cell tracking at rates of 2 μm/s and achieving resolutions of ∼70 and ∼20 nm, respectively (Hess et al., 2007; Nelson et al., 2014; Schneider et al., 2015). Another important aspect of performing live-cell imaging and viral-tracking is to avoid hindering the natural behavior of viruses by the usage of bulky labeled antibodies. Researchers have developed different approaches using click-chemistry (Wang et al., 2013; Sakin et al., 2017), SNAP tag (Eckhardt et al., 2011; Malkusch et al., 2013) or FIAsH (Lelek et al., 2012) to label *in situ* the proteins with small dyes to not hamper biological processes.

On the other hand, SRM samples can be combined with other microscopy techniques such as EM, AFM, or confocal in a method called correlative microscopy (Kim et al., 2015; Hauser et al., 2017). Correlative light and electron microscopy (CLEM) is a group of powerful multimodal imaging techniques applicable in biological research (Hodgson et al., 2018; Andrian et al., 2020). In brief, it consists in the combined application of different microscopies, thus conjoining the ability to select molecular features by fluorescent labels with fluorescence microscopy (FM), to increase the resolution by SRM and to obtain the subcellular context provided by the high-resolution of EM. Hence, it would be possible to resolve better the different viral models with a great deal of detail. Indeed, this approach was implemented in several viral studies, combining dSTORM and AFM (Dahmane et al., 2019), iPALM and SEM (Engelenburg et al., 2014; Pedersen et al., 2019), dSTORM and SEM (Kim et al., 2015) or STED and fluorescence correlation spectroscopy (FCS) (Chojnacki et al., 2017).

Finally, the increased popularity of super-resolution studies on viruses has inspired researchers to develop tools to analyze super-resolution data like VirusMapper, an open ImageJ tool to resolve nanostructures and create 2-D models from SRM data on viruses (Gray et al., 2016, 2017) in combination with several optimized protocols to study single viral particles with SRM (Gray and Albrecht, 2019; Pereira et al., 2019).

All in all, within the last decade SRM has been the protagonist of outstanding research in terms of viral structure characterization. Here we want to review the viral studies performed with different SRM techniques and their relevance, pointing out how versatile and powerful these techniques are: different viruses can be studied, all the steps of the cycle of infection could be researched and antivirals could be identified properly. Therefore, these studies could inspire future assays to unveil the viral mechanisms of COVID-19 among others.

## Virus Characterization With Super-Resolution Microscopy

**FIGURE 1:**
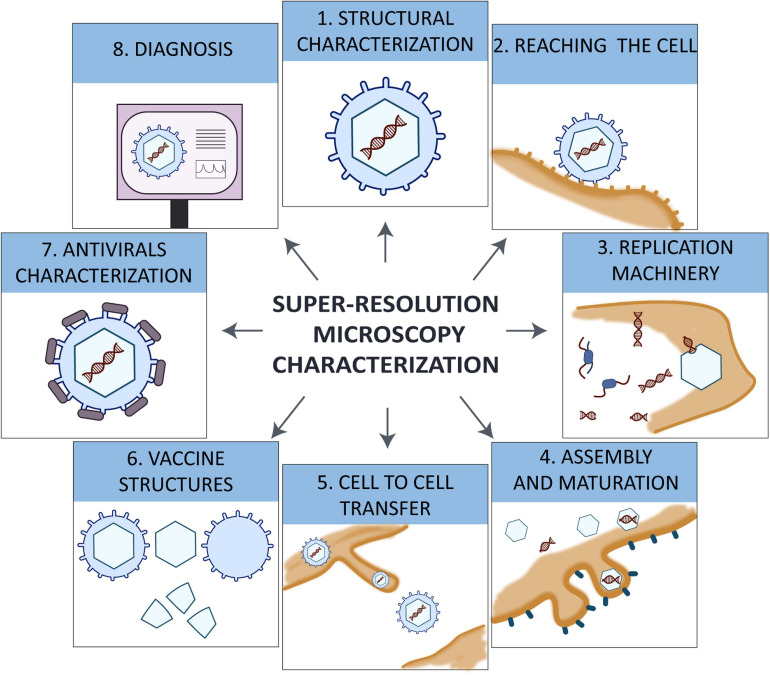
Diagram of all viral steps studied with super-resolution microscopy STED, SIM, and SMLM.

Viral life cycle requires several steps to fulfill the synthesis of new viral particles. The viral particle that resides in the environment interacts with target cells by receptor-ligand interaction, triggering the internalization of the virus to replicate the genetic material, to produce viral proteins and to assemble newly produced viral particles using the machinery of the host. Each step is essential for the viral production and needs to be studied and understood in detail to develop antivirals and mechanisms to control pandemics (Figure 1). Using SRM it is possible to have a better understanding of the mechanisms that several viruses use to spread.

### Structural Characterization

A crucial step toward the characterization of viral mechanisms of infection, and thus the blockage of viruses with antivirals, is the structural analysis of the virus particle itself. Understanding the protein distribution on the capsid and envelope proteins when the particle is isolated would help to understand the mechanisms of infection and redistribution of proteins once the virus interacts with the cell. With SRM it is possible to resolve the distribution of external proteins of several viruses, identifying and quantifying single clusters of proteins along the viral structure. Herein we summarize the state of structural characterization for different types of viruses using STORM, STED, PALM and the combination of some of them.

Herpesvirus is a dsDNA virus enclosed in a capsid and in tegument protein layers and wrapped inside an envelope composed of a lipid membrane with glycoproteins. Performing 2-color STORM on Herpes simplex virus type-1 (HSV-1), Laine et al. (2015) managed to identify the distribution of three different proteins of the tegument of HSV-1 (V16, pUL37, and VP1/2) in relation with the envelope protein (gD). With the information obtained from SMLM they were also able to develop a model-based analysis to measure the layers of each protein and the virion architecture (Laine et al., 2015). Furthermore, studies with STED microscopy also described the distribution of gH/gL envelope glycoproteins in single, double or multiple ring-shape clusters along the viral structure, complementing the virion architecture information (Beilstein et al., 2019).

Human immunodeficiency virus (HIV-1) is an enveloped positive ssRNA virus and one of the main threats against humans due to its capacity to escape from the immune system and “hide.” The viral particle undergoes a structural change during maturation, during which the proteins reorganize and the size increases. The shape and size of immature and mature HIV-1 viral particle were distinguished with PALM and STED, where immature virions showed a rounded shape (40 nm × 53 nm width × length) while mature virions were conical (62 nm × 106 nm), being possible also to track the morphological changes between both states (Lelek et al., 2012; Hanne et al., 2016a).

**FIGURE 2:**
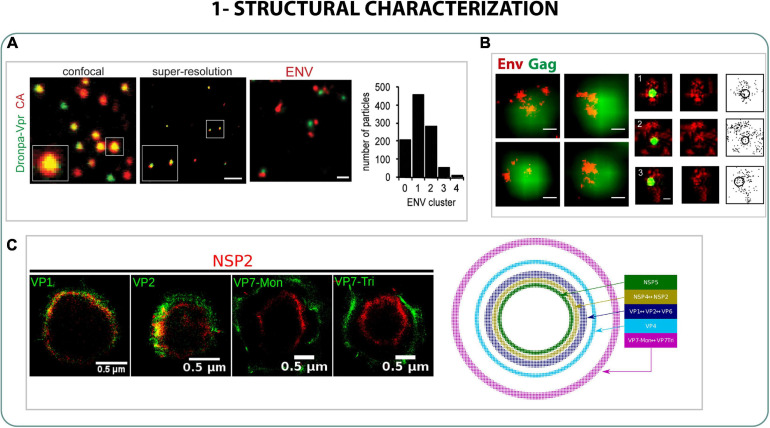
Structural characterization. **(A)** Dual color PALM/STORM and confocal images of HIV-1 virions labeled with Dronpa-Vpr (green) and CA (red). Scale bar 1 μm. Right image and graph: Super-resolution image of Env (red) on Dronpa-Vpr particles (green) and quantification of Env clusters on 1,000 HIV-1 particles. Scale bar 200 nm (Lehmann et al., 2011). **(B)** STORM and PALM on HIV-1 proteins expressed on plasma membrane (right) or on immature HIV-1 particles (left). Green is PALM (Gag) and red is STORM (Env). Scale bar 100 nm (Muranyi et al., 2013). **(C)** Rotavirus SRM 3B images of different proteins of the viroplasm. Right: model of distribution of viral components (Garcés Suárez et al., 2019).

Moreover, using 2-color PALM and STORM against immobilized HIV-1 virions it was possible to visualize the distribution along the particle of proteins of the inner structure (Gag), the matrix (MA) and envelope (Env) and also to quantify the structural sizes inside the viral particle. In mature HIV-1 particles the protein Env was found in one or two individual clusters per virion, accumulating around Gag clusters at the periphery of the viral structure (Figures 2A,B) (Lehmann et al., 2011; Muranyi et al., 2013). The main matrix MA and capsid proteins were also localized by dSTORM and cryo-EM in relation with the viral protein Vpr, suggesting that the capsid protein and MA also undergo a morphological change once the viral particle interacts with the cell triggered by binding to CD4 receptors prior to internalization (Pereira et al., 2012; Pham et al., 2015).

The structure of the Rotavirus, a dsRNA virus that mainly produces gastroenteritis disease, was also characterized using a SRM technique called “Bayesian Blinking and Bleaching” (3B). Garcés Suárez et al. (2019) analyzed the relative distribution of seven different proteins in the viroplasm of the rotavirus, and developed a model with the spatial distribution of all the proteins in the virosomes. Using the model, they observed the arrangement of the seven proteins in five different concentric layers with highly organized organelles (Figure 2C) (Garcés Suárez et al., 2019).

Lastly, vaccinia virus (VACV) or smallpox virus, an enveloped dsDNA virus of ∼270 nm width, was studied with dSTORM. Gray et al. (2019) were able to localize the cluster distribution of the binding protein (D8) and an entry fusion complex protein (EFC-L1), proving that both formed clusters along the viral particle, and are found on some specific regions of the virus like the apical and base areas. By localizing the cluster formation of both proteins in mutant VACV they were also able to identify the roles of other proteins of the EFC (A21, A28, G9, and O3) in the cluster distribution and thus, demonstrate how essential they are for the entry of the virus (Gray et al., 2019).

The distribution of the proteins involved in the recognition, attachment and cell entry is crucial for some antivirals development like multivalency architectures approaches (Bhatia et al., 2016), where the distribution of the ligands must be precise and fitting the distribution of the viral receptor. SRM could help reveal the information needed to acknowledge the viral structure characterization.

### Reaching the Cell

**FIGURE 3:**
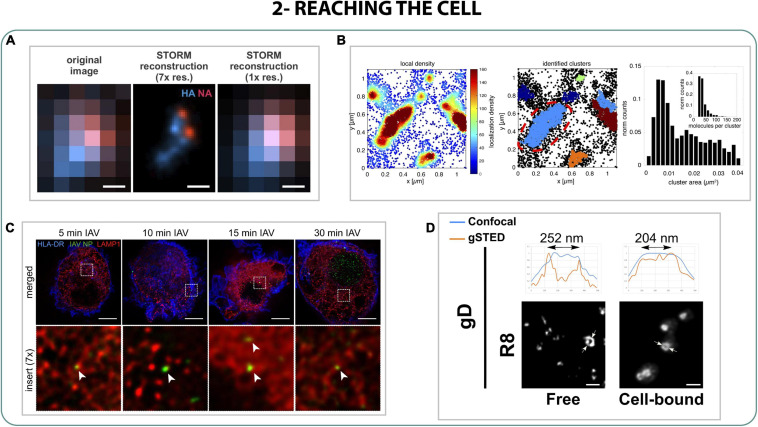
Reaching the cell. **(A)** 2-color STORM of HA (blue) and NA (red) on influenza filamentous virions. Scale bar 200 nm (Vahey and Fletcher, 2019). **(B)** STORM density-based localization analysis of small sialic acid clusters on cells (Sieben et al., 2020). **(C)** STED studies of IAV (green) trafficking through late endosomes (red) in dendritic cells from 5 to 30 min post exposure. Scale bar: 5 μm (Baharom et al., 2017). **(D)** STED images of herpesvirus glycoprotein gD in free or cell-bounded virions. Scale bar 500 nm (Beilstein et al., 2019).

Once the viral particle arrives to the cell it interacts with cell receptors, triggering conformational changes to enter and start the infection. However, in most cases the virus needs to overcome different barriers in order to arrive to target cells. This is the case of influenza A virus (IAV) that has to penetrate into the mucus barrier of the lungs to reach the underlying epithelium, where it infects. The role of the enzyme neuraminidase (NA) of influenza in this process has been deeply studied *in vitro* (Cohen et al., 2013; Yang et al., 2014) but to understand how NA interferes with the mucus cleavage in detail, Vahey and Fletcher (2019) studied with 2-color STORM the organization of hemagglutinin protein (HA) and NA proteins on virions. It was seen that NA is enriched and organized in small clusters in the apical part of the virion, while HA is slightly anti-correlated in the opposite region of the virion. This polarization is linked to a more unidirectional mobile behavior that could be translated to an efficient mucus penetration (Figure 3A) (Vahey and Fletcher, 2019).

Right after IAV reaches the target cell, the protein receptor HA binds to the sialic acid of the membrane, activating several factors that allow the internalization of IAV. Thanks to a combination of dSTORM, PALM, STED, and single-virus tracking (stpPALM), it was possible to quantify and characterize the cluster distribution on the membrane of two of the factors involved in the virus-cell binding of IAV: attachment factors (AF) and epidermal growth factor receptor (EGFR). It was observed that AF were distributed along the membrane in big and small clusters from 10 to 50 nm of length, while EGFR was organized only in small clusters overlapping AF in a non-random way, proving the connection between the attachment factors that IAV uses to infect and that EGFR uses in the binding process (Figure 3B) (Sieben et al., 2020).

Finally, subsequently to the binding to sialic acid (SA), IAV internalizes into the host cell by several endosomal pathways. Using STED on dendritic cells it was possible to characterize the trafficking of the virus through the early and late endosomes (5 and 15 min, respectively), following the viral particles from the entrance to the nucleus import (Figure 3C), describing also how antiviral interferon (RIG-1 and IFITM3) plays an important role blocking that initial step of internalization (Baharom et al., 2017; Sánchez-Aparicio et al., 2017; Kummer et al., 2019).

In the case of other viruses like Herpes HSV-1, the glycoprotein gD plays a crucial role in binding to cell membrane receptors while other glycoproteins (gh/gL and gB) interfere with the membrane fusion to host cells. Experiments performed with STED showed that the binding to the receptors of the membrane triggered a change in the glycoproteins’ conformation, especially in gD. Once the viral particle bound to the cell, gD changed its distribution from small clusters to a high-density ring-shape distribution on the surface along the structure, presumably to finish the maturation of the virus and help the cell entry (Figure 3D)(Beilstein et al., 2019).

### Genetic Replication Machinery Tracking

Right after the entry of the viral particle into host cells, genetic material needs to replicate in the cytoplasm or in the nucleus. DNA viruses such as adenovirus or RNA viruses like influenza possess a mechanism to deliver their genetic material into the nucleus of the host cells by surpassing cytoplasmic controls that normally destroy exogenous genetic material.

STED and confocal studies were used in adenovirus using modified nucleotides by click-chemistry to track viral genome in the cytoplasm. Wang et al. (2013) proved that once the viral DNA started to be separated from the capsid (90 min post infection), the DNA decondensed and occupied more area in both the cytoplasm and the nucleus, measuring a surprisingly low efficiency of nuclear import (6–48%) (Wang et al., 2013).

**FIGURE 4:**
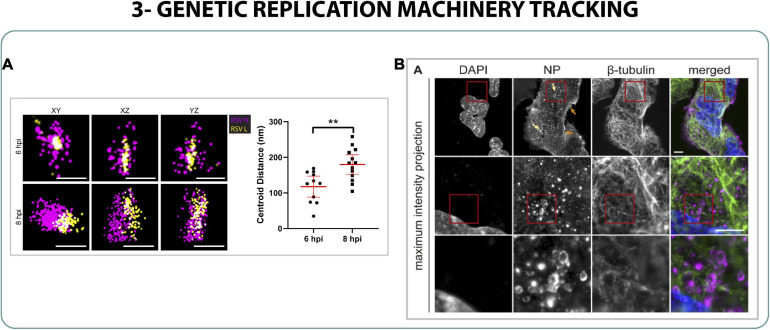
Genetic replication machinery tracking. **(A)** STORM imaging and distance measurement of RSV ribonucleocapsid proteins L (yellow) and N (magenta) at 6 and 8 h post infection (hpi). Scale bar 200 nm (Blanchard et al., 2020). **(B)** Expansion/light sheet of cells infected with live attenuated influenza NPs (Mascheroni et al., 2020). A Welch t-test was performed, where *n* = 10 and **p < 0.01.

On the other hand, to study the influenza replication one could focus on the nucleoproteins (NPs) since they are interacting with the vRNA of the virus and are involved in the viral replication (Portela and Digard, 2002; Krug and Fodor, 2013). For this purpose, Mascheroni et al. (2020) used a non-conventional super-resolution approach combining expansion microscopy and light sheet microscopy. Expansion microscopy (Chen et al., 2015) is a technique that uses mechanical forces to physically expand the cell in order to enlarge the structures that are closer to the diffraction limit, which, in combination with light sheet microscopy, could allow the identification of NPs located in ∼250 nm vesicular structures surrounding the nucleus of the cell. It was presumed that the ribonucleoprotein-genome (RNP) complex starts to assemble inside the membranes of the endosome machinery in the cytoplasm (Figure 4B) after being released from the nucleus in a process that involved enlargement of the nucleopores via caspases, characterized by SIM (Mühlbauer et al., 2015; Mascheroni et al., 2020).

Moreover, using a similar click-chemistry approach to label nucleotides as mentioned above, Peng et al. (2014) tracked with multicolor STORM and PALM the journey of HIV-1 genome inside cells. HIV-1 replicates its genetic material in the cytoplasm, but it is integrated inside the genetic material of the host in the nucleus. With SMLM they could calculate the time frame of nuclear import (24–48 h) and all the localizations of the capsid, reverse transcription complex and integration complex along the internalization process (Peng et al., 2014).

Finally, the negative-sense RNA of the respiratory syncytial virus (RSV) needs the ribonucleocapsid of the virus to be protected in the cytoplasm, to be able to replicate and to translate. Using dSTORM it was possible to characterize the distribution of the proteins L and N of the ribonucleocapsid during the cycle of infection of RSV, understanding the roles of those proteins within the RNA of the virus and highlighting the rearrangement of the complex L-N during replication between 6 and 8 h post infection (Blanchard et al., 2020) (Figure 4A).

### Assembly – Maturation Virus

Right after the proteins have been translated and the genetic material replicated, the newly synthetized viral material needs to find its way to the cell membrane, so it can assemble and get released from the cell. One of the most studied viruses in this field is HIV-1, particular the understanding of the distribution of proteins Env and Gag on budding areas.

**FIGURE 5:**
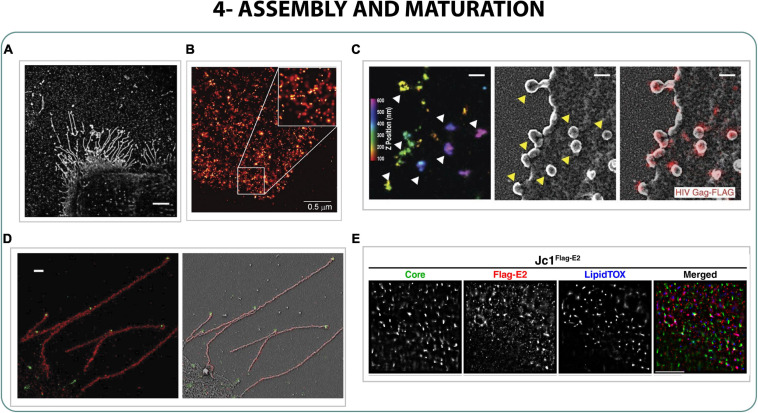
Assembly and maturation. **(A)** STORM images of Udorn influenza filaments on infected cells. Scale bar 5 μm (Kolpe et al., 2019). **(B)** PALM image of COS-7 expressing Gag HIV-1 protein (Betzig et al., 2006). **(C)** Correlative iPALM-SEM of Gag protein on cells infected with HIV-1. Scale bar 250 nm (Engelenburg et al., 2014). **(D)** Correlative 3D STORM and SEM of the proteins M1 (red) and vRNP (green) of influenza filaments of Udorn strain on infected cells. Scale bar 500 nm (Kim et al., 2015). **(E)** 3-color STORM of core, flag-E2 and lipid droplets of Hepatitis C (Eggert et al., 2014).

By performing correlative STED-confocal it was possible to confirm that Env clusters were recruited in surrounding areas of Gag assembly sites, where Gag interfered in the distribution of Env clusters (Sakin et al., 2017). Particularly, it was confirmed with correlative STED-FCS and by dSTORM that Gag plays a crucial role in Env cluster mobility at viral assembly sites, important for viral particle maturation (Roy et al., 2013; Chojnacki et al., 2017). On the other hand, Gag itself accumulates in budding areas forming three different patterns: small clusters (40–50 nm), medium clusters (140 nm) and patchy aggregates of bigger sizes (Figures 5B,C), characterized by dSTORM, PALM and STED applying a coordinate-based cluster analysis (Betzig et al., 2006; Eckhardt et al., 2011; Gunzenhäuser et al., 2012; Malkusch et al., 2013).

The budding and release of HIV needs also several cellular factors to reorganize the membrane and allow the viral release. Tetherin is an interferon that interferes with the release of HIV-1 from the cell membrane, being crucial to control the infectivity of the virus. Using multicolor PALM and STORM, researchers observed the presence of tetherin, Gag and Env protein on assembly sites on the membrane, quantifying as well the clusters that those proteins form on the membrane in order to understand the model of association of the tetherin proteins and HIV-1 (Lehmann et al., 2011; Grover et al., 2013). Another important factor is the endosomal sorting complex required for transport (ESCRT), needed to mediate the virus release. The smart combination of different techniques helped to resolve the arrangement of the proteins of the complex ESCRT at the budding area of HIV-1 using either CLEM with iPALM and SEM, iPALM or 2 color STORM co-localizing the budding areas with Gag and ESCRT, helping in the development of an assembly model for the virus budding. Using this model, it was possible to characterize the need of the recruitment of ESCRT complex in the neck of assembly areas of HIV-1 to facilitate the excision of virions and to enhance tetherin recruitment (Grover et al., 2013; Engelenburg et al., 2014; Bleck et al., 2014; Prescher et al., 2015).

Finally, additional important cellular components in HIV budding and formation are tetraspanins CD9 and CD81, membrane remodeling factors in viral infections (Nydegger et al., 2006, p. 1; Jolly and Sattentau, 2007; Grigorov et al., 2009). Correlative dSTORM/AFM was performed to have a nanoscale map of the interactions between Gag and CD9/CD81. Both factors were located mostly in small clusters, with an average diameter of 70 and 81 nm, respectively, on the tip of the protrusion and especially concentrated on enriched Gag areas, suggesting their role in membrane curvature for budding (Dahmane et al., 2019).

In conclusion, with SRM it was possible to develop a model of several factors involved in the budding and release of HIV-1 as well as to identify the positions of the proteins Gag and Env, crucial for the viral particle formation.

Regarding studying assembly and maturation models with SRM in other viruses, STORM was widely used to characterize the viral protein distribution in herpes HSV-1, Influenza, Nipah virus and hepatitis.

As explained in structural characterization, herpes HSV-1 consists of three layers of proteins and lipids (capsid, tegument, and envelope). Using 2-color dSTORM it was possible to monitor the assembly and maturation of these three layers by following the protein VP26 (capsid) from its assembly in the nucleus to the cytoplasm and localizing the protein gM (envelope) surrounding the nuclear membrane, waiting for the tegument to be formed. On the contrary, tegument proteins were always present on the viral particles during its assembly regardless the formation stage (Laine et al., 2015).

Some strains of IAV can form filaments on the surface of infected cells or directly rounded viral particles. Furthermore, the role of filament formation of influenza strain Udorn was characterized with dSTORM and correlative STORM-SEM. These filaments showed the presence of the proteins M1, M2, and HA homogeneously along the filament and RNPs mainly in condensed budding areas in the apical part of the filaments, suggesting the role of influenza filaments in the formation of viral particles from the apical part of the filament (Figures 5A–D) (Noda et al., 2006; Kim et al., 2015; Kolpe et al., 2019). Besides, multicolor-live FPALM and STORM was applied to the characterization of the pathway that influenza uses to express its proteins on the plasma membrane of infected cells. It was observed how HA domains arrived from the Golgi to the plasma membrane through the action of phosphatidylinositol (PIP2) and actin, forming cluster-associated structures in domains between 30 and 120 nm width (Hess et al., 2007; Itano et al., 2012; Gudheti et al., 2013; Curthoys et al., 2019).

Finally, three-color 3D dSTORM was performed to study the distribution of two main proteins of hepatitis virus C (core and E2) compared to the distribution of the lipid droplets (assembly sites) during the assembly process, not finding a clear co-localization of the proteins within the droplets and neither a specific pattern of distribution inside them (Figure 5E), suggesting the need of correlative approaches to fully understand the mechanism of viral formation in infected cells (Eggert et al., 2014). Similarly, a scholastic Nipah virus model was determined by STORM, where the Matrix protein M formed dome-like clusters on the membrane independently of the incorporation of the Glycoprotein G or the fusion protein F (Liu et al., 2018).

Other SRM approaches to study different virus such as Zika virus or Vesicular stomatitis virus G protein (VSVG) required multidisciplinary microscope techniques. TEM, SEM, and SR-SIM were performed to characterize the internal distribution of the Zika virus in infected cells, describing the association of the main protein of ZIKV and the endoplasmic reticulum (ER) where it is suggested that immature virus accumulates inside, modifying the structure of the ER (Caldas et al., 2020). It is possible to study the dynamics of *in vivo* proteins and molecules by improving PALM with single-particle tracking in a new technique named sptPALM (Manley et al., 2010), used to follow the homogeneous distribution of vesicular stomatitis virus G protein (VSVG) and its dynamics along the membrane (Manley et al., 2008).

The approach used to study the viral assembly and budding depends strongly on the type of virus, its mechanism of maturation and the factors involved, thus selecting the right SRM technique is key to characterize the model and obtain the big picture in the viral assembly step.

### Cell to Cell Transfer

In the cycle of infection of some viruses it is common to find mechanisms of transferring viral particles from cell to cell without the release of the viral particle into the environment. Since these mechanisms are quite unknown, the study of viral cell-to-cell transfer with SRM could be interesting for the design of antivirals and blocking systems against viruses.

**FIGURE 6:**
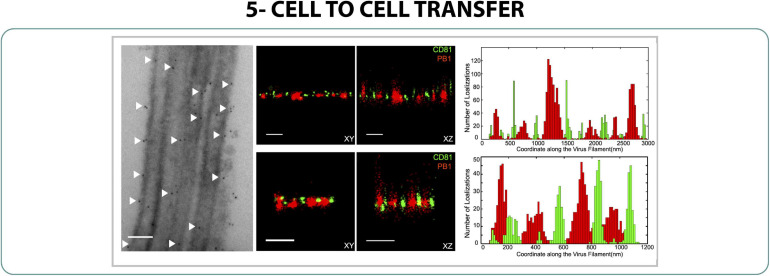
Cell to cell transfer. EM **(left)** and 2-color 3D STORM **(middle)** of CD81 (green) and PB1 (red) on filaments of Influenza. Scale bar 500 nm (He et al., 2013).

As commented before, some strains of influenza can form filaments on their surface when the virus is isolated from clinical samples. It is thought that these filaments not only produce new viral particles but also play an important role in the cell-to-cell transmission, delivering genetic material to new nearby host cells (Cifuentes-Muñoz et al., 2018). Understanding this mechanism of transmission is difficult due to the small size of the filaments and, though it has been explored by expansion microscopy (Kumar et al., 2017), there are still unknowns about the main mechanism of cell-to-cell transmission. It is thought that CD81, a tetraspanin, plays an important role in the process, therefore the distribution of CD81 and viral polymerase (PB1) along the filaments was characterized with 3D 2-color dSTORM, observing that both proteins formed separated and individual clusters along the filament formation (Figure 6) (He et al., 2013). It was suggested that this organized pattern could have an implication on the cell-to-cell transmission role as happens with HIV-1 (Martin et al., 2005).

The main characteristic of the HIV-1virus is its capacity to infect CD4+ T cells from intact dendritic cells (DC) in a process called “trans infection” (Rinaldo, 2013). Identifying the mechanism behind this process is essential to understand the spread of the virus and thus the infection, since HIV-1 takes advantage of the DC antigen presenting mechanism. The receptor CD169 has been identified as the mayor capture receptor involved in the *trans* infection mechanism (Izquierdo-Useros et al., 2012). Using FPALM, Akiyama et al. (2015) localized the distribution of CD169 on DCs with HIV-1, describing an accumulation of both CD169 and HIV-1 in pocket-like compartments of 800 nm to 1 μm in depth inside DCs connected to the surface. It was hypothesized that HIV-1 hides in these compartments, avoiding the endocytic pathways of DCs that could degrade the virus, preventing also from its antigen presentation to T cells (Akiyama et al., 2015). Similar studies have been done on astrocytes and macrophages, possible reservoirs of HIV-1 with STED, to characterize how they transmit the virus to other cell types (Russell et al., 2017).

Finally, herpesviruses are famous for causing quiescent infections in the peripheral nervous system nearby the initial host cell, transporting viral cargos along the axon to spread to new host mucosal cells (Smith G.A. et al., 2001). Studying herpes virus simplex 1 (HSV-1) with SIM, Scherer et al. (2020) could observe how viral membrane components US7, US8, and US9 form a complex at the Golgi network that could recruit kinesin-3 motors and thus transport them through the axon of the neuron by vesicle transportation to new host cells (Scherer et al., 2020).

In general, SRM are a compilation of different techniques that, with the right conditions, could allow the study of all the steps that form part of a viral infection, unveiling the pathways that viruses employ to conquer host cells.

## Antivirals Study by Super-Resolution Microscopy

Viral characterization with SRM does not stop when the viral particle is released and fully active, viral studies embrace more features such as development of new antivirals and vaccines and the detection and identification of viruses in waste and samples (Figure 1). The potential use of super-resolution for characterization of viral structures with high sensitivity and precision could help in the development of new drugs and detection systems against different types of viruses.

### Virus-Like Particles as Vaccine Candidates

Virus-like particles are empty viral structures that can mimic the shape and size of the original virus and have been proposed for several purposes such as universal vaccines and drug delivery vectors (Kang et al., 2012; Zdanowicz and Chroboczek, 2016).

**FIGURE 7:**
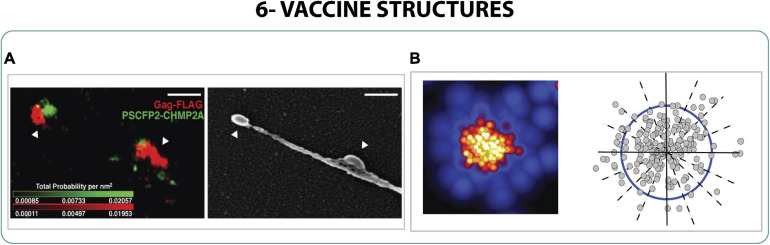
Vaccine structures. **(A)** Correlative 2-color iPALM and SEM of two virus-like particles during budding from the cell. Scale bar 250 nm (Engelenburg et al., 2014). **(B)** PALM localization and quantification of the morphology of Gag on HIV-1 VLPs (Gunzenhäuser et al., 2014).

The production of VLPs needs to be optimized and monitored to identify the critical formation steps to produce successful VLPs; accordingly, the study with SRM could help the identification of VLP structures. Studying the HIV-1 VLP formation with sptPALM, correlative iPALM and SEM and PALM with molecular counting, the molecular composition of VLPs was described and the crucial role of Gag in VLP formation was characterized. Not only was Gag the only viral protein needed to produce VLP, but also it induced the curvature of the membrane creating the protuberance for the budding (Figures 7A,B). The viral particles created had a width between 160 and 220 nm and were found in specific areas of the membrane where Gag was immobilized (Manley et al., 2008; Engelenburg et al., 2014; Gunzenhäuser et al., 2014; Pedersen et al., 2019).

Finally, a new approach was suggested with an intermediate resolution between confocal and super-resolution by using Hyvolution microscope to obtain a final resolution of 140 nm. With this microscopy the production of VLP of HIV-1 could be monitored and analyzed from the budding to the release. Also, the total amount of VLPs produced could be quantified while evaluating the quality in harvested supernatants, information that was used to improve their clinical applications (González-Domínguez et al., 2020a, b).

### Antivirals and Monoclonal Antibodies

Antivirals are usually tested *in vitro* using different assays like hemagglutination inhibition assay, cell–cell fusion assay, western blot or enzymatic approaches which suffer from several limitations such as lack of specificity of antibodies and reagents or a deficient sensitivity in some assays (Ustinov et al., 2017; Rumlová and Ruml, 2018). Therefore, for a thorough understanding of the implications of antivirals on viral structures and their action, a different approach is needed such as SRM, which could allow the identification of the antiviral effect on the viral structure for further optimization of the drug.

**FIGURE 8:**
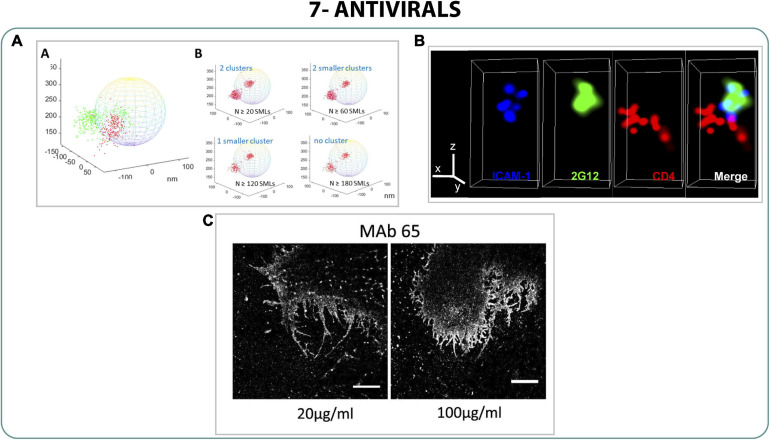
Antivirals. **(A)** 3D iPALM imaging of Env (red) and SER5 (Green) projected onto a 200 nm diameter model sphere representing a viral particle (Chen et al., 2020). **(B)** STORM images of the monoclonal antibody 2G12 (green) with the virion (blue) and CD4 receptor (red) (Mengistu et al., 2015). **(C)** STORM images of filaments of influenza treated with two different concentrations of a monoclonal antibody MAb 65. Scale bar 5 μm (Kolpe et al., 2019).

SERINC5 (serine incorporator 5) is a proven antiviral against HIV produced by the host cell that is inserted into the virions inhibiting the fusion to the host cell membrane, increasing also the sensitivity to neutralizing antibodies and antiviral drugs (Rosa et al., 2015; Sood et al., 2017; Pye et al., 2020). Since the mechanism of action of SERINC5 was unclear, Chen et al. (2020) used dSTORM and iPALM to characterize the interaction between SERINC5 and Env and the subsequent disruption of the virion. They described the cluster distribution of Env protein and demonstrated that SERINC5 disrupted that Env cluster formation without direct binding (Figure 8A). This impediment to produce clusters by disturbing the mobility of Env does not allow the maturation of the viral particle. Accordingly, as Chojnacki et al. (2012, 2017) described with STED and correlative STED-FCS, this avoids the fusion between virus-host cell membranes, leading to a decrease of the infectivity of the virus (Chen et al., 2020).

Monoclonal antibodies are interesting antivirals due to their high specificity against concrete viral epitopes, but the distribution of those epitopes on the surface of viral particles is crucial to block the virus infection. To understand whether the epitopes of the protein gp120 of HIV-1 were exposed during the viral particle attachment to cells, Mengistu et al. (2015) performed 3D STORM analysis to compare the exposure of several neutralizing epitopes on the viral particle whilst interacting with the CD4 cells (Figure 8B) (Mengistu et al., 2015).

Moreover, the effect of monoclonal antibodies on influenza virus morphology was studied with dSTORM. Kolpe et al. (2019) studied the filament formation of Udorn strain under the treatment of different monoclonal antibodies against the protein M2, describing abnormal filaments on infected cells treated with monoclonal antibodies and correlating the defective filament formation with a decrease of infectivity of the virus (Figure 8C) (Kolpe et al., 2019).

### SRM for Diagnostics

Lastly, sensitive, rapid and unequivocal identification and detection of viral presence in different samples is essential to control pandemics and allow a fast characterization of the infection. Several studies proposed SRM approaches with TIRF, machine learning or STED to be able to identify and classify groups of viruses from the same sample, increasing the effectiveness of high-throughput assays.

To increase the sensitivity of the test used for diagnosis of viruses such as SARS-CoV-2, Shiaelis et al. (2020) developed a fast diagnosis system (5 min) using labeling, immobilization and single-particle imaging in TIRF of individual viruses, connected to a machine-learning analysis. The system was tested using three influenza strains and one human coronavirus (hCoV), viruses that cannot be distinguished in fluorescence microscopes due to their similar size and shape. The system achieved high accuracy values (95%), and identified and classified the virus strains presented in a sample without purification or amplification (Shiaelis et al., 2020).

**FIGURE 9:**
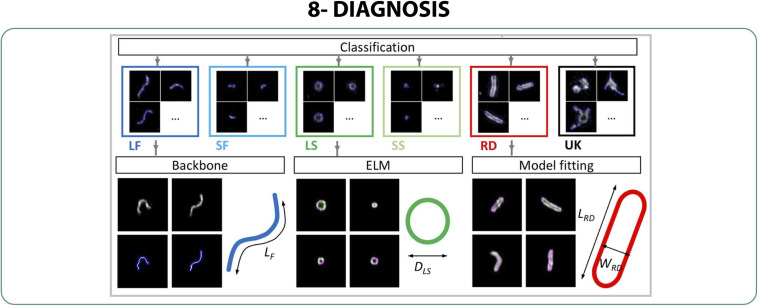
Diagnosis. Workflow of the automated detection and classification method using TIRF-SIM to classify NDV shape in high-throughput (Laine et al., 2018).

An improved system using high-throughput imaging combining TIRF-SIM and supervised machine learning algorithms allowed the morphological classification of a population of Newcastle Disease Virus (NDV), providing enough resolution to discern between filamentous, spherical and rounded structures fast and in large amounts (Figure 9). It was also indicated to be efficient in the classification of influenza vaccinia candidates in non-purified harvested fluid in order to discriminate which strain successfully produced live attenuated influenza virus (Laine et al., 2018).

Lastly, molecularly imprinted polymers (MIPs) have been studied for detection and purification of viruses that could bind with high affinity and specificity. Using STED, Gast et al. (2020) characterized the binding affinity and stability of adenovirus on the detection beads MIP, concluding that 30–40 adenovirus particles could bind in a mechanically stable way, proving that this detection system is robust, versatile, stable, and cost-efficient (Gast et al., 2020).

## Concluding Remarks

Viruses are always present in our everyday life: from the Rhinovirus that gives us a simple cold to COVID-19 or HIV-1 that have created sanitary crises around the world, viruses are invisible hazards that need to be studied in detail with powerful techniques. The incredibly small size of these organisms, together with their fast mutations and adaptation, has prompted the need to use single-particle techniques with high sensitivity and resolution to characterize the small changes in their structures and identify the key factors involved in their infection cycle. SRM has been deemed as an ideal technique to characterize viruses due to its high specificity, high resolution, live-cell imaging and 3D multicolor images that fit perfectly with the requirements for new viral studies. The last 10 years have been crucial in the development of SRM studies to characterize viral infection with all types of super-resolution techniques and different approaches to fulfill the gaps of information in current viral models. This approach has been covered in other reviews that focus only on the study of HIV-1 with SRM (Müller and Heilemann, 2013; Hanne et al., 2016b; Chojnacki and Eggeling, 2018), and on the study of influenza replication by SRM and advanced quantitative microscopy (Touizer et al., 2021).

Here, we described the most promising viral studies using SRM to prove how each step of the viral cycle and antiviral design could be studied with a super-resolution approach, resolving models impossible to be resolved with conventional microscopy. Studying the viral particle itself resolved the distribution of key proteins of different viruses, thus understanding how they mature once they are released from infected cells. Furthermore, from the cell interaction to the assembly, proteins could be monitored and quantified, identifying the clusters of proteins and their role in the infection of viruses. Additionally, SRM could be implemented in the relevant study of the molecular changes triggered by viral infection inside cells, such as the reorganization of the endoplasmic reticulum given by Zika infection (Long et al., 2020), the modification of the membrane due to the filament formation of influenza (Kolpe et al., 2019) or the spatial re-arrangement of ESCRT machinery by HIV-1 (Bleck et al., 2014).

It is also possible to study the rational design of antivirals with SRM, combining high-throughput methods with the resolution gained with SRM to improve the viral inactivation and vaccine production. With this broad approach including different types of viruses, we wanted to highlight the flexibility of SRM in terms of sample nature, for example studying viruses with or without capsid, with RNA, DNA, or VLPs with only one protein on their structure.

Nevertheless, SRM has some drawbacks that have to be taken into account such as the need of expensive and delicate microscopes with powerful lasers, optimization of sample preparation, long exposure times and high technical skills. Yet, in the last years more and more improvements have been applied to the SRM field with the development of new dyes, computational improvements and the creation of new algorithms to increase the speed and sensitivity with lower costs, which within the following years should help surpass the disadvantages of SRM (Lehmann et al., 2016; Ma et al., 2017; Mazidi et al., 2018; Moore and Legant, 2018; Ouyang et al., 2018; Mahecic et al., 2019).

These improvements could make SRM more accessible for future assays to unveil viral mechanism and the smart antiviral design for viruses such as COVID-19, facilitating fast and precise viral models.

## Author Contributions

MA-R wrote the manuscript. MA-R, SP, and LA edited the manuscript and provided the conception of this review. All authors contributed to the article and approved the submitted manuscript.

## Conflict of Interest

The authors declare that the research was conducted in the absence of any commercial or financial relationships that could be construed as a potential conflict of interest.

## Abbreviations

Cryo-EM: cryo-electron microscopy
dsDNA: double-stranded deoxyribonucleic acid
EM: electron microscopy
HIV-1: human immunodeficiency virus type-1
HSV-1: herpes virus type-1
IAV: influenza A virus
NP: nucleoprotein
PAINT: points accumulation for imaging in nanoscale topography
PALM: photoactivated localization microscopy
RNP: ribonucleoprotein
RSV: respiratory syncytial virus
SIM: structured illumination microscopy
SMLM: single-molecule localization microscopy
SRM: super-resolution microscopy
ssRNA: single-stranded ribonucleic acid
STED: stimulated emission depletion
STORM: stochastic optical reconstruction microscopy
VACV: vaccinia virus
VLP: virus-like particle.
